# Chromosome-level assembly of the synthetic hexaploid wheat-derived cultivar Chuanmai 104

**DOI:** 10.1038/s41597-024-03527-2

**Published:** 2024-06-22

**Authors:** Zehou Liu, Fan Yang, Cao Deng, Hongshen Wan, Hao Tang, Junyan Feng, Qin Wang, Ning Yang, Jun Li, Wuyun Yang

**Affiliations:** 1 Crop Research Institute, Sichuan Academy of Agricultural Sciences, Chengdu, China; 2 Environment Friendly Crop Germplasm Innovation and Genetic Improvement Key Laboratory of Sichuan Province, Chengdu, China; 3 Key Laboratory of Wheat Biology and Genetic Improvement on Southwestern China, Chengdu, China; 4 Key Laboratory of Tianfu Seed Industry Innovation, Chengdu, China; 5 Biotechnology and Nuclear Technology Research Institute, Sichuan Academy of Agricultural Sciences, Chengdu, China; 6 The Key Laboratory of Animal Disease and Human Health of Sichuan Province, College of Veterinary Medicine, Sichuan Agricultural University, Chengdu, China; 7 Departments of Bioinformatics, DNA Stories Bioinformatics Center, Chengdu, China

**Keywords:** Genome, Plant breeding

## Abstract

Synthetic hexaploid wheats (SHWs) are effective genetic resources for transferring agronomically important genes from wild relatives to common wheat (*Triticum aestivum* L.). Dozens of reference-quality pseudomolecule assemblies of hexaploid wheat have been generated, but none is reported for SHW-derived cultivars. Here, we generated a chromosome-scale assembly for the SHW-derived cultivar ‘Chuanmai 104’ based on PacBio HiFi reads and chromosome conformation capture sequencing. The total assembly size was 14.81 Gb with a contig N50 length of 58.25 Mb. A BUSCO analysis yielded a completeness score of 99.30%. In total, repetitive elements comprised 81.36% of the genome and 122,554 high-confidence protein-coding gene models were predicted. In summary, the first chromosome-level assembly for a SHW-derived cultivar presents a promising outlook for the study and utilization of SHWs in wheat improvement, which is essential to meet the global food demand.

## Background & Summary

Common wheat (*Triticum aestivum* L., 2*n* = 6*x* = 42, AABBDD) is a natural amphiploid derived from the intergeneric cross between *T. turgidum* subsp. *durum* (Desf.) Husn., a cultivated allotetraploid (2*n* = 4*x* = 28, AABB), and *Aegilops tauschii* Coss., a diploid goat grass (2*n* = 2*x* = 14, DD). The genetic diversity among common wheat cultivars has been drastically reduced owing to bottlenecks resulting from polyploidy, domestication, and modern plant breeding. The decline in genetic diversity can be counteracted by direct hybridization between common wheat and *A. tauschii*^1,2^, or through hybridization between common wheat and synthetic hexaploid wheats (SHWs) developed by crossing tetraploid wheat and *A. tauschii*^3^.

The primary objective of the direct hybridization method is to augment genetic diversity specifically for the D genome in common wheat, addressing a crucial concern in wheat breeding, because significantly lower genetic diversity values characterize this genome compared with the A and B genomes^4^. However, the diminished genetic diversity resulting from the bottlenecks also affects the A and B genomes. Consequently, the utilization of SHW lines enables the diversity of all three subgenomes of common wheat to be enhanced. This approach facilitates the direct transfer of genes/loci for traits of interest from diploid and tetraploid to hexaploid wheat.

To date, the International Maize and Wheat Improvement Centre (CIMMYT) has developed more than 1200 SHW lines^3^. Since the introduction of more than 200 SHW accessions from CIMMYT in 1995, four SHW-derived cultivars, namely, Chuanmai 38, 42, 43, and 47, have been raised and cultivated, which have been widely used in wheat breeding as elite parents in China. Subsequently, a number of secondary SHW-derived cultivars have been developed and released, including Chuanmai 104, developed from the cross of Chuanmai 42 and Chuannong 16. Chuanmai 104 is an important high-yielding wheat cultivar grown in Southwest China in recent years. The maximum yield of Chuanmai 104 attains 10,947 kg/ha under the humid and predominantly cloudy climate of the Sichuan Basin in Southwest China^5^. Chuanmai 104 is becoming a cornerstone breeding parent of wheat in China. Furthermore, China is among the main countries that are exploiting the advantages of SHW lines as genetic resources, especially in Southwest China^3^. The increasing utilization of SHW worldwide is indicative of the success of such an approach, which will gradually become an effective means of overcoming the bottleneck of wheat breeding.

Considering previous studies based on SHWs, a major potential limiting factor is the limited genetic resources and lack of reference-quality pseudomolecule assemblies (RQAs)^6^. Chapman *et al*. integrated whole-genome sequencing and genetic mapping to assemble and ordered contigs of the SHW cultivar W7984^7^. However, given the short reads generated by next-generation sequencing (NGS), and the lack of chromosome conformation capture sequencing or chromosome isolation via flow sorting, the assembly was only 9.1 Gb, which was substantially less than the estimated 15 Gb size of the hexaploid wheat genome^6^. Although single-nucleotide polymorphism (SNP) genotyping arrays are relatively simple and inexpensive, a limitation is that only the variants pre-selected for inclusion on the array can be analyzed. Consequently, if the SNP panels were designed using common wheat genome assemblies, they would lack sufficient representation of variants in the target gene pools, and thus assessment of useful variation in SHW and derivative germplasm would be challenging. More recently, reduction in costs have meant that RQAs and large-scale whole-genome resequencing are feasible and affordable for SHWs.

**Fig. 1:**
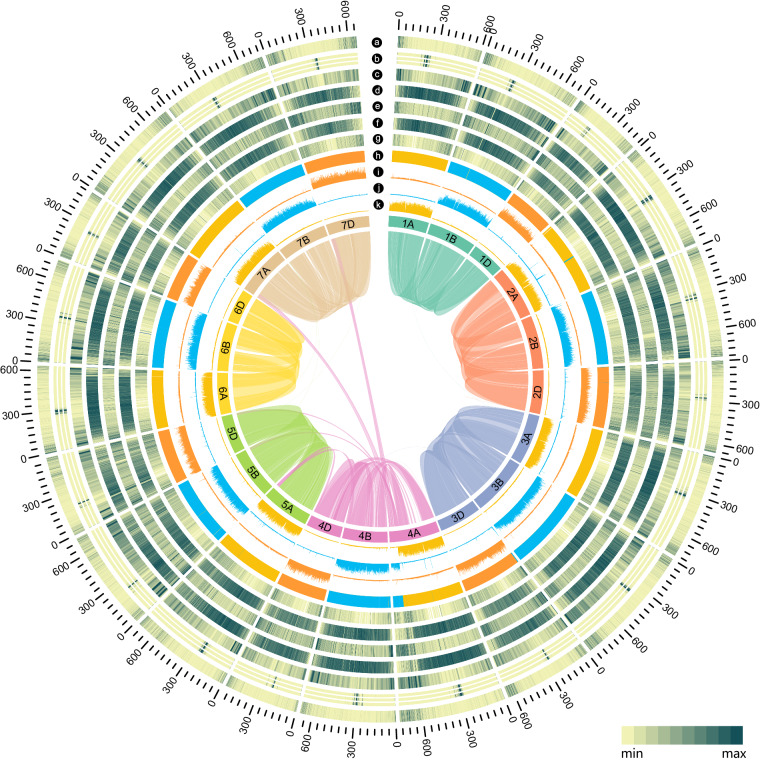
Overview of Chuanmai 104 chromosome-scale assembly. (**a**) Distribution of the *A. tauschii* clone A6-10 subtelomeric tandem repeat sequence (GenBank Accession AY249980.1). (**b**) Distribution of the *A. tauschii* clone 6C6-3 (GenBank Accession AY249981.1) and 6C6-4 (GenBank Accession AY249982.1) and *T. monococcum ssp. aegilopoides* clone BAC TbBAC5 (GenBank Accession DQ904440.1) and TbBAC30 (GenBank Accession EF624064.1) centromere-specific tandem repeat sequences. (**c**) Distribution of the non-coding gene density. (**d**) Distribution of the transposable elements’ density. (**e**) Distribution of the tandem repeat density. (**f**) Distribution of the long terminal repeat density. (**g**) Distribution of the high-confidence protein-coding gene density. (**h**) Distribution of the significant enrichment of subgenome-specific k-mers identified by SubPhaser (gold colour for A, blue for B, and orange for D). (**i**) density distribution of the D subgenome-specific k-mer set. (**j**) density distribution of the B subgenome-specific k-mer set. (**k**) density distribution of the A subgenome-specific k-mer set. Links between chromosomes are collinearity blocks, which are coloured according to the homologous chromosomes. All the densities were calculated using sliding windows (window size: 1Mbp, step size: 1Mbp), except the density distribution of the non-coding genes, which use a window size of 10Mbp and a step size of 1Mbp for smoother visualization.

In the current study, we first generated a chromosome-level assembly for Chuanmai 104 (Fig. 1), based on an integrated approach including PacBio HiFi sequencing reads and chromosome conformation capture sequencing. The final Chuanmai 104 genome assembly consisted of 14.81 Gb with a contig N50 of 58.25 Mb, a contig N90 of 8.41 Mb, and a longest contig of 422.27 Mb (Table 1). Among previously published hexaploidy wheat assemblies, seven of the 21 chromosomes in the Chuanmai 104 were the longest (Table 2). The long terminal repeat (LTR) Assembly Index (LAI)^8^ of the Chuanmai 104 genome assembly was 15.17, 14.64, and 10.85 for A subgenome, B subgenome, D subgenome respectively, and for each chromosome, the LAI values ranges from 10.21 to 15.71 (Table 3). Benchmarking universal single-copy orthologs (BUSCO) analysis yielded a completeness score of 99.30%, which was comparable with that of common wheat genomes and notably higher than that of the SHW cultivar W7984 (Table 1). Repeats comprised 81.36% of the sequences with a predominance of retrotransposons, which accounted for 62.96% of the sequences (Table 4). In total, 122,554 high-confidence and 136,431 low-confidence protein-coding gene models were predicted (Table 5); this number was similar to that for the common wheat Chinese Spring (Table 1). The high-quality Chuanmai 104 genome assembly generated in this study provides a reference genome for SHW-derived cultivars, and offers a promising outlook for the study and utilization of SHW genetic resources in wheat improvement, which is essential to meet the global food demand.

**Table 1:** The summary results of the SHWs (CM104 and W7984) and common wheats (Fielder, Kariega, Attraktion, Renan, CS) genome assemblies.

Genomic features	CM104	W7984	Fielder	Kariega	Attraktion	Renan	CS
Total size (Gbp)	14.81	8.51	14.70	14.68	14.68	14.26	14.57
Longest contig (Mbp)	422.27	0.10	171.22	158.43	113.94	15.12	3.53
# Contigs	6,817	2,856,525	5,202	5,511	4,995	12,877	306,274
contig N50 (Mbp)	58.25	0.01	20.68	26.66	16.70	2.16	0.34
contig L50	64	257,497	180	134	239	1,958	12,219
contig N90 (Mbp)	8.41	0.001	3.83	4.45	3.20	0.60	0.03
contig L90	353	1,218,830	824	643	1,019	6,645	59,196
BUSCO completeness	99.30%	93.30%	99.30%	99.30%	99.40%	99.20%	99.30%
# HC genes	122,554	NA	116,480	116,838	NA	109,543	126,244

NA: not available. CS: Chinese Spring.

**Table 2:** The chromosomes lengths of selected representative hexaploid wheat genomes.

Chromosome	CM104	Fielder	Kariega	Attraktion	Renan	Chinese Spring
Chr1A	609,472,862	608,979,116	613,662,638	605,966,608	593,930,347	598,660,471
Chr1B	727,830,234	720,972,993	717,109,572	703,076,930	702,775,664	700,547,350
Chr1D	500,453,425	501,257,520	504,659,958	495,911,329	494,594,617	498,638,509
Chr2A	788,446,683	804,602,427	794,474,755	796,169,439	792,837,209	787,782,082
Chr2B	805,159,295	808,121,247	817,712,742	779,372,321	812,232,696	812,755,788
Chr2D	662,232,259	649,118,519	662,526,948	665,561,653	661,835,603	656,544,405
Chr3A	761,348,578	758,906,661	760,111,594	757,165,295	750,337,041	754,128,162
Chr3B	875,847,286	861,141,126	864,624,966	852,704,148	854,463,248	851,934,019
Chr3D	646,472,733	642,382,296	633,282,846	623,698,249	623,248,023	619,618,552
Chr4A	767,264,397	759,893,476	769,810,128	745,048,881	749,950,614	754,227,511
Chr4B	690,023,074	689,766,370	701,857,263	677,947,850	673,746,810	673,810,255
Chr4D	534,597,632	531,462,149	534,651,777	524,289,323	520,815,567	518,332,611
Chr5A	726,476,980	714,517,032	715,684,684	726,838,826	712,547,961	713,360,525
Chr5B	727,782,148	717,288,350	726,425,509	701,430,346	703,299,309	714,805,278
Chr5D	585,259,613	586,345,039	584,285,409	584,133,940	569,771,178	569,951,140
Chr6A	633,963,269	626,266,972	623,890,083	622,677,745	620,176,429	622,669,697
Chr6B	742,272,111	738,085,275	738,041,677	745,712,656	717,542,863	731,188,232
Chr6D	506,848,534	505,809,789	507,261,758	490,622,797	493,761,083	495,380,293
Chr7A	753,207,242	759,124,079	755,457,679	748,850,018	746,502,734	744,491,536
Chr7B	761,653,245	751,612,808	767,912,069	753,856,519	752,612,656	764,081,788
Chr7D	660,255,776	653,055,523	659,687,352	643,890,519	648,661,963	642,921,167

**Table 3:** Statistics of number of contigs, LAI and non-coding RNAs on each chromosome in Chuanmai 104.

ID	# contigs	LAI	miRNA	rRNA	snoRNA	snRNA	tRNA
Chr1A	21	15.32	1,492	1,328	246	14	585
Chr1B	70	14.65	2,515	4,277	263	38	597
Chr1D	5	11.48	1,108	2,682	174	21	574
Chr2A	23	15.40	1,982	99	183	37	617
Chr2B	53	14.94	3,175	122	184	50	622
Chr2D	10	11.09	1,577	83	117	41	594
Chr3A	13	15.11	1,762	106	133	33	662
Chr3B	73	14.74	3,117	136	235	39	845
Chr3D	17	10.33	1,321	92	97	46	693
Chr4A	41	14.35	1,827	143	178	162	687
Chr4B	65	14.25	2,211	114	190	16	428
Chr4D	5	10.21	951	96	122	14	502
Chr5A	15	15.71	1,727	1,068	239	30	614
Chr5B	63	14.77	2,985	2,159	362	83	535
Chr5D	12	11.77	1,267	1,629	174	117	618
Chr6A	12	15.47	1,412	79	189	53	452
Chr6B	73	14.72	2,439	688	305	71	600
Chr6D	10	10.81	991	68	197	56	482
Chr7A	23	14.93	1,929	94	158	60	641
Chr7B	68	14.39	2,900	130	195	79	564
Chr7D	16	10.42	1,367	184	136	63	651

**Table 4:** The statistics for the repeats in the Chuanmai 104 genome.

Repeats types	A (%)	B (%)	D (%)	A + B + D (%)
Total	83.37	82.81	80.57	81.36
**Retrotransposons**	66.72	65.23	58.85	62.96
LTR	65.87	64.23	58.24	62.08
*LTR/Copia*	17.65	16.35	16.79	16.67
*LTR/Gypsy*	46.19	45.19	39.17	43.10
*LTR/Unknown*	2.01	2.68	2.27	2.30
LINE	0.08	0.50	0.07	0.29
*LINE/L1*	0.08	0.49	0.07	0.28
SINE	0.76	0.49	0.54	0.59
**DNA-transposons**	15.00	15.49	19.72	16.36
Helitron	0.08	0.11	0.10	0.17
TIR	14.90	15.37	19.61	16.17
*TIR/CACTA*	11.66	12.91	16.96	13.39
*TIR/CMC-EnSpm*	0.26	0.20	0.15	0.21
*TIR/Harbinger*	0.52	0.44	0.50	0.48
*TIR/hAT*	0.04	0.06	0.08	0.06
*TIR/hAT-Ac*	0.01	0.02	0.01	0.01
*TIR/hAT-Tag1*	0.02	0.03	0.01	0.02
*TIR/Mariner*	1.21	0.88	1.04	1.03
*TIR/MULE-MuDR*	0.12	0.12	0.16	0.15
*TIR/Mutator*	0.41	0.31	0.34	0.35
*TIR/PIF-Harbinger*	0.02	0.02	0.03	0.02
*TIR/TcMar-Stowaway*	0.13	0.08	0.10	0.10
*TIR/unknown*	0.48	0.28	0.22	0.33
*Unknown*	0.02	0.01	0.01	0.01
**Tandem Repeats**	3.06	4.72	5.18	4.38
**Unknown**	0.95	1.02	0.84	1.00

Only the repeat types with percentage larger than 0.01% were listed. The bold text indicates the class, the regular text indicates the superfamily, while the italic text indicates the family.

**Table 5:** Statistics of gene structural and functional annotation.

Types		sources	# genes
Structural annotation	de novo	AUGUSTUS	179,081
		GeneMark-ET	566,794
		GeneID	736,750
	transcriptome	Iso-Seq	281,737
	homology	*H. vulgare* Morex	14,693
		*T. urartu*	17,252
		*Ae. speltoides*	24,962
		*Ae. tauschii*	14,644
		*T. turgidum subsp. dicoccoides*	19,228
		*T. turgidum subsp. durum*	57,595
		*T. aestivum* Chinese Spring	205,435
	final	high -confidence (HC)	122,554
		low-confidence (LC)	136,431
Functional annotation of HC genes		InterPro	108,423
		KEGG	50,374
		NR	121,301
		SwissProt	76,076
		Overall	121,695

## Methods

### Plant material, DNA extraction, and sequencing

The SHW-derived cultivar Chuanmai 104 was kindly provided by Wuyun Yang (Crop Research Institute, Sichuan Academy of Agricultural Sciences). The plants used for sequencing were grown in a growth chamber with a controlled environment of 20 degree Celsius under a 12 h light/12 h dark photoperiod for 2 weeks. Genomic DNA (gDNA) was extracted from seedling leaf tissues using the cetyltrimethylammonium bromide method. Three methods were applied for DNA quantification and quality testing, including (i) NanoDrop 2000 spectrophotometer (Thermo Fischer Scientific), (ii) gel electrophoresis and (iii) Qubit fluorometer (Invitrogen). Total DNA was purified by AMPure PB beads (Pacific Biosciences, CA, USA; PN 100-265-900). High-quality gDNA (≥10 μg, ≥100 ng/μl) was prepared for the next step of library construction. PacBio single-molecule real-time (SMRT) bell library preparation was performed using the SMRTbell® Express Template Prep Kit 2.0 (Pacific Biosciences, CA, USA; PN 101-853-100) in accordance with the manufacturer’s instructions. The library was prepared for sequencing with a 30 h movie on the Sequel IIe system (Pacific Biosciences) by the Berry Genomics Corporation (Beijing, China). Totally, we generated 668.43 Gb bases (~45X) with 40,999,150 CCS reads from 20 SMRT cells.

Chromosome conformation capture (Hi-C) sequencing of Chuanmai 104 was performed using the protocol of Peng *et al*.^9^. In brief, 2–4 g tender leaves from the plants used for genome sequencing were harvested and stored in liquid nitrogen, and then the Hi-C libraries were prepared and sequenced on the MGISEQ-2000 platform by BGI (Wuhan, China). Samples were cut into pieces of ca. 2 cm^2^, and transferred to 50 ml tubes containing 15 ml of ice-cold nuclear isolation buffer (NBE) with 2% formaldehyde, followed by vacuum infiltration (400 mbar) and incubation with a supplemented cross-linking agent for 1 h. Cross-linking was quenched by adding 2 M glycine to a final concentration of 0.125 M with incubation for 5 min under vacuum, followed by fixation on ice. Then, the fixed leaf pieces were washed three times with sterile Milli-Q water, ground in liquid nitrogen and subjected to nucleus isolation. The isolated nuclei were purified, checked for quality and quantity and digested with 100 units of *DpnII*. The next steps were Hi-C specific, including marking the DNA ends with biotin-14-dATP and performing blunt-end ligation of the cross-linked fragments. After ligation, cross-linking was reversed by overnight incubation with proteinase K at 65 °C. Biotin-14-dATP was further removed from non-ligated DNA ends using the exonuclease activity of T4 DNA polymerase. DNA was purified by phenol:chloroform (1:1) extraction, precipitated and washed as previously described. The purified DNA was physically sheared to a size of 300–600 bp by sonication and was size-fractionated using standard 2% agarose gel electrophoresis to obtain fragments in the range of 300–600 bp. The fragmented ends were blunt-end repaired and A-tailed, followed by purification through biotin-streptavidin-mediated pulldown. PCR amplification was conducted using 12–15 cycles to enrich the ligation products. Totally, we generated more than 2 Tb bases (>135 X) with 6.69 Gb read pairs.

For full-length transcriptome sequencing, we collected pooled sample for Chuanmai 104, which comprised whole plant organs except for roots from seed germination to the three-leaf stage, shoots at the seedling stage, and leaves, stems, ears, and seeds from the heading to the late-filling stages. Total RNA was isolated using TRIzol Reagent in accordance with the manufacturer’s instructions (Thermofisher). The RNA purity and raw contamination were first assed by Nanodrop 2000 (Thermo Fischer Scientific), and then the RNA Integrity Number (RIN) and concentration were further assessed by an Agilent 4200 (Agilent Technologies). High-quality RNA (2 μg, 300 ng/μl) was prepared for the next step of library construction. PacBio SMRT bell library preparation was performed using the SMRTbell® Express Template Prep Kit 2.0 (Pacific Biosciences) in accordance with the manufacturer’s instructions. The library was prepared for sequencing with a 30 h movie on the Sequel IIe system (Pacific Biosciences) by the Berry Genomics Corporation. Totally, we generated 186.35 Gb bases with 2,283,790 polymerase reads from one SMRT cell. The final 46,130,981 subreads range from 51 bp to 241,082 bp, with a mean and N50 value of 4,039.55 bp and 4,561 bp respectively.

### Genome assembly

The PacBio HiFi CCS reads were assembled using hifiasm^10^ (v0.16.1, with default parameters). The Hi-C reads were incorporated using Juicer tools^11^ (v1.6) and EndHiC^12^. In brief, preprocessing of the Hi-C reads was performed with juicer.sh^11^ (parameter: -s *DpnII*). The output file corresponding to the Hi-C contacts with duplicates removed and mapping quality values larger than 30 was generated as input for EndHiC^12^. These result files were plotted to visualize the Hi-C map and for manual curation, and were used to generate the final assembly (21 pseudomolecules and one unanchored pseudomolecule). The NCBI Foreign Contamination Screen (FCS)^13^ was used to identify and remove contaminant sequences (adaptors and organelles) in genome assemblies. Totally, the FCS identified total 754 contaminant fragments, including one adaptor fragment and 753 mitochondrial fragments, and all these contaminants are located on the unanchored pseudomolecule and were masked.

### Validation of genome assemblies

Genome sizes were estimated using three algorithms (gce^14^, GenomeScope2^15^, and findGSE^16^) with different *k*-mer sizes. The quality and completeness of the genome assemblies were assessed by merqury^17^, which uses a reference-free, *k*-mer-based approach, and BUSCO^18^ (v5, poales_odb10), which is based on evolutionarily informed expectations of the near-universal single-copy orthologous gene content. LTR assembly index (LAI)^8^ that evaluates assembly continuity using LTR-RTs were calculated.

### Subgenome assignment, validation, and nomenclature

To assign each chromosome to each linkage group and apply the corresponding nomenclature in Chinese Spring, SubPhaser^19^, a robust allopolyploid subgenome phasing method based on subgenome-specific *k*-mers, was used. To validate the correctness of the subgenome assignment, a reference-guided strategy based on subgenome homology was also used to distinguish the subgenomes. We mapped the Chuanmai 104 genome to the Chinese Spring genome using mashmap^20^ (-f map–perc_identity 90 -s 1000000). Then, the alignments were plotted and manually checked. This procedure successfully categorized the 21 chromosomes into three homologous groups. The nomenclature system for Chinese Spring chromosomes was adopted for naming of the homologous groups (1–7) of the Chuanmai 104 genome.

### Repeat annotation

Tandem repeats of all lengths were annotated with TandemRepeatsFinder^21^ v4.09 using the default parameters (Match Mismatch Delta PM PI Minscore MaxPeriod: 2 7 7 80 10 50 500). LTR_FINDER^22^ (v1.05) and LTR_harvest^23^ (v1.5.10) were used for long terminal repeat (LTR) identification, and the results were processed with LTR_retriever^24^ (v2.8) to generate a species-specific LTR library. The species-specific LTR libraries, wheat transposable element (TE) sequences from ClariTeRep (https://github.com/jdaron/CLARI-TE), and plant TE sequences from Repbase^25^ were merged to generate the TE library. Transposons were detected and classified by a homology search against the combined TE library. The program Vmatch (http://www.vmatch.de/), a fast and efficient matching tool suitable for large and highly repetitive genomes, was used for this computationally intensive task with the following parameters: identity ≥ 70%, minimal hit length 75 bp, and seed length 12 bp (exact command line: -d -p -l 75 -identity 70 -seedlength 12 -exdrop 5).

### Non-coding gene annotation

Noncoding RNAs (ncRNAs), including miRNAs, small nuclear RNAs, rRNAs and regulatory elements, were identified using the Infernal^26^ (version 1.1.2) program to search against the Rfam^27^ database (v14.8). The rRNAs, and tRNAs were further identified using RNAmmer^28^ (version 1.2) and tRNAscan-SE^29^ (v1.3.1) respectively.

### Protein-coding gene annotation

Gene model prediction was performed following the method described by Mascher *et al*.^30^, with minor modifications, which integrated transcriptomic data, protein homology, and *ab initio* prediction. (1) First, isoform sequencing (Iso-Seq) data were mapped to the genome using minimap2^31^ (v2.17-r941; parameters: -ax splice -uf –secondary = no -C5). The redundant isoforms were further collapsed into transcript loci using cDNA_Cupcake (https://github.com/Magdoll/cDNA_Cupcake) (parameter: –dun-merge-5-shorter). TransDecoder (v5.5.0, https://github.com/TransDecoder/TransDecoder) was used to predict protein sequences among the transcripts. (2) For protein homology evidence, we projected the gene structures from Triticeae species, comprising *Ae. tauschii*, *T. turgidum* subsp*. dicoccoides*, *T. turgidum* subsp*. durum*, *T. aestivum* Chinese Spring, *T. urartu*, *Ae. speltoides*, and *Hordeum vulgare* Morex, onto the Chuanmai 104 genome using liftoff^32^ with default parameters. (3) We produced *ab initio* gene predictions using AUGUSTUS^33^ (v3.4.0), GeneMark-ET^34^ (v4.38), and GeneID^35^ (v1.4). In brief, AUGUSTUS^33^ gene prediction was performed using a model specifically trained from the software and a hints file generated using the previously mentioned Iso-Seq predictions. GeneMark-ET^34^ was used with the option -ET, and the intron coordinates were calculated using the above-mentioned Iso-Seq alignments. GeneID^35^ was run with a model specifically trained from the software (-GP taestivum.param). We used EVidenceModeler^36^ (EVM; v1.1.1) to integrate all of the gene evidence from transcriptomics, protein alignments, and *ab initio* predictions.

Protein-coding gene models from EVM were classified as high-confidence or low-confidence according to criteria used by the International Wheat Genome Sequencing Consortium, with minor modifications^37^. In brief, protein-coding gene models were considered as ‘complete’ when start and stop codons were present. A comparison with PTREP^38^ (the database of hypothetical proteins deduced from the nonredundant database of TEs within the TREP database), UniPoa^39^ (Poaceae database of annotated proteins from the UniProt database), and UniMag^40^ (validated Magnoliophyta proteins from SwissProt) was performed using DIAMOND^41^ (v2.0.9; parameters: -e 1e-10 –query-cover 80–subject-cover 80). Gene candidates were classified using the following criteria: a high-confidence gene model was ‘complete’ with a hit in the UniMag^40^ database and/or in UniPoa^39^ but not PTREP^38^; the remaining gene models were classified as low-confidence genes.

Functional assignments of the predicted protein-coding genes were obtained with BLAST^42^ by aligning the coding regions to sequences in public protein databases, including the trEMBL^40^, RefSeq^43^., and SwissProt^40^ databases. The putative domains and GO^44^ terms of the predicted proteins were identified using the InterProScan^45^ program. The putative orthologs in the KEGG^46^ database were identified using KoFamScan^47^.

## Data Records

The HiFi reads, Iso-seq reads, and Hi-C reads that were used for the Chuanmai 104 genome assembly have been deposited in the NCBI Sequence Read Archive with accession number SRP488123 and under BioProject number PRJNA1070409^48^. The HiFi reads, Iso-seq reads, and Hi-C reads were also deposited in the National Genomics Data Centre (NGDC) with BioProject ID PRJCA022052 (https://ngdc.cncb.ac.cn/bioproject/browse/PRJCA022052). The genome assembly has been deposited at GenBank under the accession JBBIFV000000000^49^. The genome assemblies and annotations have also been deposited at FigShare^50^ with doi number 10.6084/m9.figshare.25282654.

## Technical Validation

**Fig. 2:**
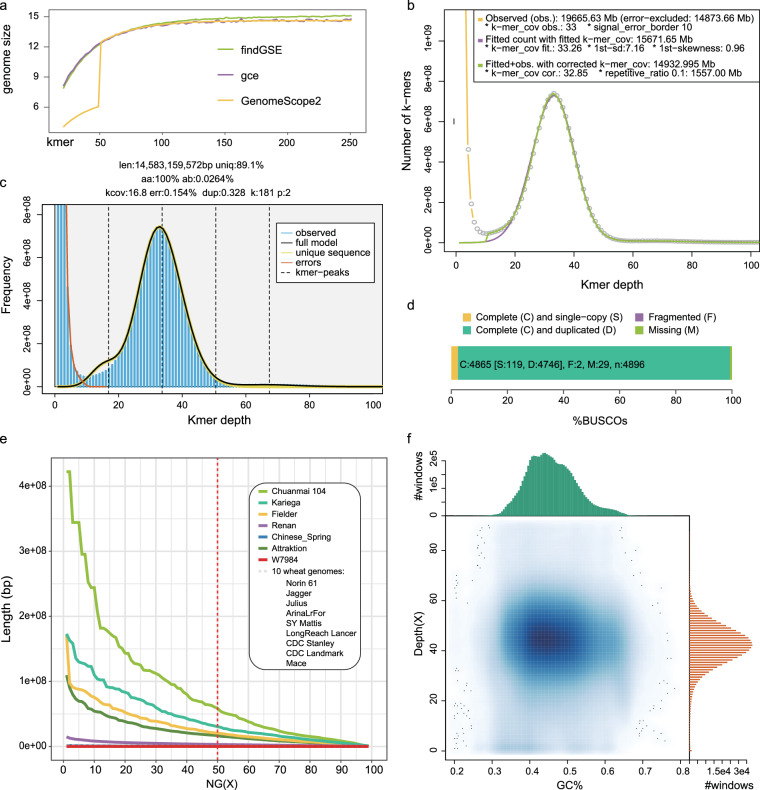
Validations of Chuanmai 104 genome assemblies. (**a**) Genome sizes estimated using different algorithms with different K-mer sizes. (**b,c**) examples of genome size estimated by findGCE (K = 181, **b**) and GenomeScope2 (K = 181, **c**) respectively. (**d**) Gene completeness assessed by BUSCO using the Poales dataset with a total of 4896 groups. (**e**) NGx plots for the Chuanmai 104 and other common wheat genomes. (**f**) GC content and average sequencing depth (GC-depth) plot of the Chuanmai 104 genome across every 2-kb nonoverlapping sliding window.

The assembled genome size is similar to the size estimated by different algorithms^14–16^ (Fig. 2a–c), and is significantly larger than that published previously for the SHW cultivar W7984 (Table 1). The base-level accuracy QV (consensus quality value) and *k*-mer completeness scores evaluated with merqury^17^ are 65.86 and 97.59%, respectively. The long terminal repeat (LTR) Assembly Index (LAI)^8^ of the Chuanmai 104 genome assembly was 15.17, 14.64, and 10.85 for A subgenome, B subgenome, D subgenome respectively, which are higher than the LAI values obtained for Chinese Spring (11.88, 12.51 and 9.97 for A subgenome, B subgenome, D subgenome respectively). The BUSCO^18^ score is 99.3% and only 0.7% BUSCO genes are missing (Fig. 2d). These results indicate a high completeness of the Chuanmai 104 assembly. Comparison with other common wheat genome assemblies revealed that the Chuanmai 104 NG50 value was significantly larger, implying high connectivity (Fig. 2e). The GC-depth plot (Fig. 2f) of the Chuanmai 104 genome across every 2 kb nonoverlapping sliding window showed no distinct secondary peaks, indicating that haplotype homology was adequately recognized during assembly.

**Fig. 3:**
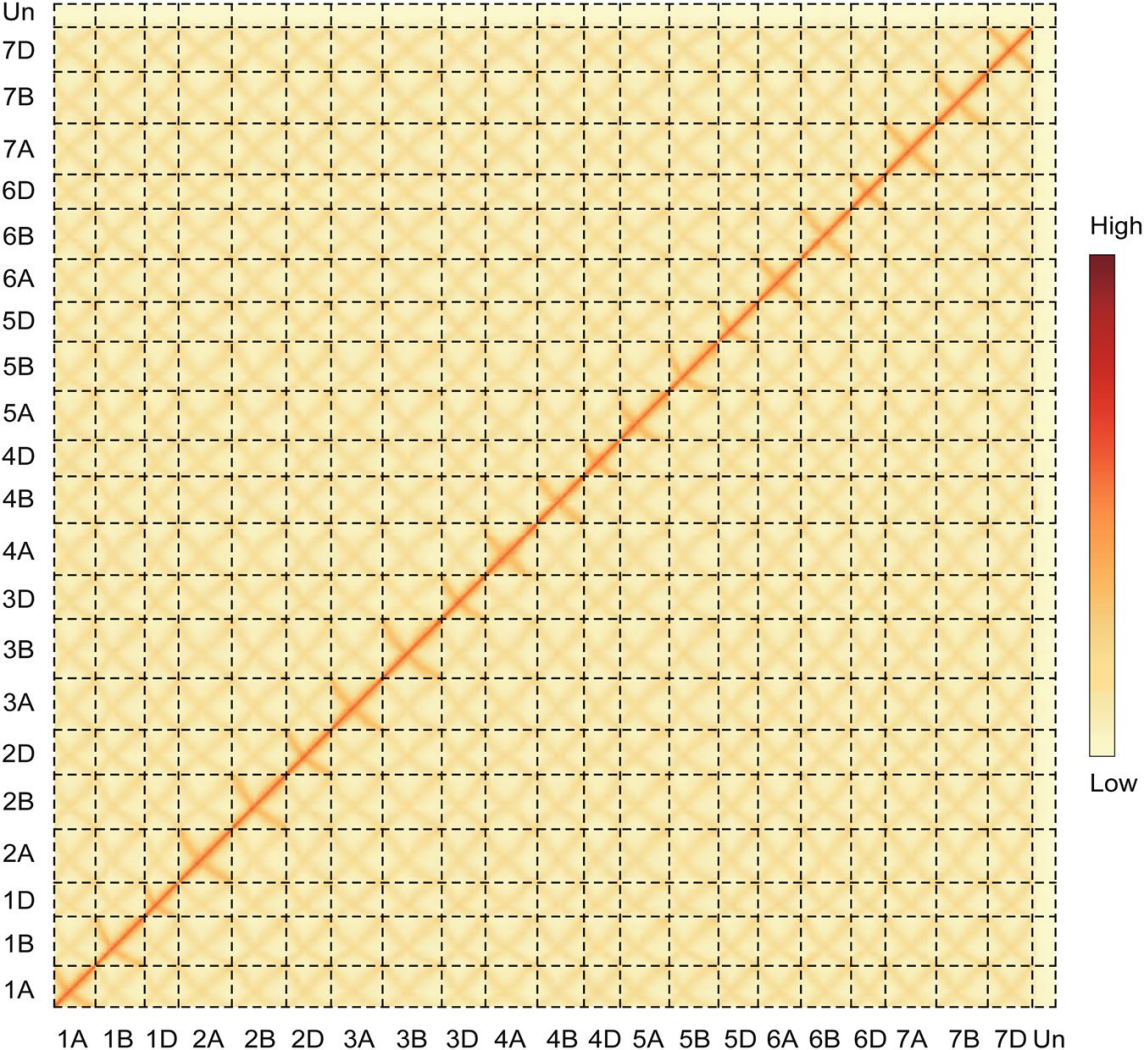
Hi-C contact maps of chromosomes. The dashed lines indicate chromosomes boundaries.

The Hi-C contact map was manually curated and assessed with Juicebox and revealed a dense pattern along the diagonal, indicating no potential mis-assemblies (Fig. 3). The anti-diagonals are typical for Triticeae genomes^51^ (Fig. 3). The distribution of the *A. tauschii* subtelomeric tandem repeat sequences (NCBI GenBank accessions: AY249980.1, AY249981.1, and AY249982.1) and *T. monococcum* subsp. *aegilopoides* centromere-specific tandem repeat sequences (NCBI GenBank accessions: DQ904440.1 and EF624064.1) indicate the completeness in these complex regions (Fig. 1a–c).

**Fig. 4:**
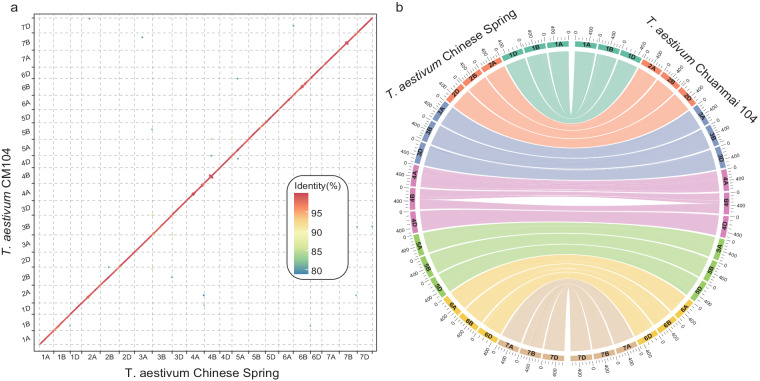
Nucleotide-level (**a**) and protein-level (**b**) synteny between the 21 chromosomes of Chuanmai 104 and Chinese Spring.

Using SubPhaser^19^, a robust allopolyploid subgenome phasing method based on subgenome-specific *k*-mers, the 21 chromosomes of the Chuanmai 104 genome were aggregated into three linkage groups (Fig. 1h–k). These groups show high synteny to chromosomes of Chinese Spring at both the nucleotide and protein levels (Fig. 4), indicating the correctness of the chromosome assembly. Moreover, these synteny results show the relative conservation of the common wheat and SHW genomes, although the sources of the subgenomes and their evolutionary history differ.

## Data Availability

All software and pipelines were executed according to the manual and protocol of published tools. No custom code was generated for these analyses.
